# The effect of level-marked mathematics tasks on students’ self-efficacy: An experimental study

**DOI:** 10.3389/fpsyg.2023.1116386

**Published:** 2023-03-17

**Authors:** Maria Herset, Mohamed El Ghami, Annette Hessen Bjerke

**Affiliations:** ^1^Faculty of Education and Art, Nord University, Nesna, Norway; ^2^Faculty of Education and International Studies, OsloMet, Oslo, Norway

**Keywords:** self-efficacy, differentiated instruction, tiered lessons, mathematics tasks, mathematics education, textbook in mathematics

## Abstract

This study investigates whether and to what extent students’ self-efficacy in mathematics is affected by level-marked mathematics tasks. An online survey with an experimental design was used to collect data from lower secondary school students in Norway (*n* = 436). The effect of level-marked mathematics tasks was measured by comparing students’ responses to tasks with no level marking with their responses to the same tasks marked as being easy, medium or difficult. The study’s design was set up carefully, featuring experimental and control groups. A Wilcoxon test showed a significant gap in students’ self-efficacy when approaching the same tasks without level marking and with difficult-level marking. In addition, a Friedman test showed that the gap between students’ self-efficacy when encountering the same task with and without level marking expanded significantly with increasing difficulty markings. This result has implications for students in terms of their mathematics learning and for mathematics teachers in terms of their future differentiation initiatives.

## 1. Introduction

The question of how to ensure high-quality mathematics experiences for all students that specifically meet their individual needs challenges teachers around the world. This challenge calls for differentiating initiatives that provide “equal opportunities to participate, and engage” (Christenson and Wager, 2012, p. 194). The purpose of differentiation is to tailor instruction so that there are “multiple options for taking in information” (Tomlinson, 2001, p. 1) to achieve an optimal learning experience and to improve self-efficacy in students (Mathiassen, 2009; NOU, 2016, p. 62).

According to Tomlinson (2001), there is a need to differentiate instruction in terms of content (what students learn), process (how they make sense of ideas and information), and product (how students demonstrate what they have learned). Here, we focus on differentiated instruction based on content and readiness by using level-marked mathematics tasks, as in tiered teaching (Pierce and Adams, 2005). We know that level-marked tasks feature in mathematics teachers’ accounts of their teaching (Brändström, 2005; Czeglédy and Szász, 2005; Eriksen et al., 2022) and are used extensively as differentiation initiatives in mathematics classrooms (Grave and Pepin, 2015). In this regard, many mathematics textbooks have a system for marking the difficulty of tasks to help students “find their way” through them (Imsen, 2020, p. 421). Mathematics textbooks have long held a strong position as the main resource for planning and executing the teaching of mathematics (Robitaille and Travers, 1992; Howson, 1995; Stein et al., 2007; Jablonka and Johansson, 2010) and recent studies have confirmed their persistent use (Dolonen et al., 2016).

An appropriate level of difficulty in mathematics is important for ensuring mastery experiences for students, and it is therefore necessary for textbooks and teachers to take differentiated instruction into account (Skaalvik and Fossen, 1995). However, there is a need to examine the interaction between students’ self-efficacy and teachers’ differentiation initiatives more closely (Herset, 2014; McNeill and Polly, 2023; Herset and El Ghami, 2022). To the best of our knowledge, no research has reported on how the extensive use of level-marked tasks affects students’ mathematics self-efficacy. Hence, since self-efficacy – a person’s “beliefs in one’s capabilities to organise and execute the courses of action required to produce given attainments” (Bandura, 1997, p. 3) – is a future-oriented construct that correlates with achievement (Pajares and Miller, 1995; Pajares, 1996), we aimed to report the results from a Norwegian study investigating the effects of level-marked mathematics tasks on students’ mathematics self-efficacy. While previous research has focused on students’ changes in self-efficacy over time, making it hard to say exactly why these changes took place (Street et al., 2022a), the current study investigates how self-efficacy is affected by level-marked tasks within a short time span (allowing no other factors to influence their change in self-efficacy, if present). In this way, this paper sheds new light on tiered teaching according to readiness.

## 2. Theoretical framework and research question

Before we examine previous research on differentiated instruction in mathematics and the role of the textbook and its use of level-marked tasks, we begin this section by providing a more detailed account of self-efficacy, its sources and its importance for mathematics learning in individuals.

### 2.1. Self-efficacy beliefs

According to Bandura (1997), self-efficacy beliefs differ in level, strength and generality. *Level* refers to whether a person perceives a given task as easy or difficult, and is a personal opinion that affects one’s choice of task or activity, one’s effort and one’s persistence (Bandura, 1997). People with low self-efficacy for accomplishing a task may avoid the task, while a more efficacious person will persist longer when encountering difficulties, with more motivation to prepare for and put effort into completing the task at hand (Schunk, 1991). Street et al. (2017) claimed that students’ perceptions of difficulty levels differ and may not reflect the actual difficulty of the task. How students perceive task difficulty is important because this perception affects their self-efficacy (Chen and Zimmerman, 2007; Street et al., 2022b).

Self-efficacy can also vary in *strength*, revealing how strong a person’s beliefs are that they can complete a given task, and *generality*, which refers to a person’s breadth of knowledge and mastery of various topics. Bandura (1997) therefore distinguished between specific self-efficacy and general self-efficacy, as self-efficacy can vary depending on the specific task, theme or subject. This was also supported by Street et al.’s (2022a) study, in which students’ self-efficacy in geometry and algebra differed. In this paper, we are mostly concerned with measuring the strength of students’ self-efficacy, while also revealing some aspects of their level of self-efficacy, as the two constructs are clearly related (Bandura, 1997).

Bandura (1997) proposed four sources as crucial in fostering self-efficacy in individuals. *Mastery experience*, which is about interpreting the results of one’s own previous attainment, was considered by Bandura (1997) to be the most powerful source, a statement repeatedly confirmed and reported in a growing body of research (e.g., Stevens et al., 2006; Usher and Pajares, 2009; Joët et al., 2011; Butz and Usher, 2015). Mastery experiences have been found necessary for students to develop and preserve expectations of mastery (Skaalvik and Skaalvik, 2018, p. 197). *Vicarious experience* is derived from observing others performing a task, which is important in building self-efficacy beliefs in individuals (Bandura, 1997). In mathematics, if students watch others who are similar to them, such as classmates, accomplishing a difficult task, it may convince them that they are able to succeed as well (Schunk, 1991). However, previous research has shown contradictory results when it comes to the relationship between self-efficacy and vicarious experience; for example, Joët et al. (2011) found no significant correlation between vicarious experience and self-efficacy, while Usher and Pajares (2009) suggested the opposite. What seems to be uncontested is that information obtained vicariously typically has a weaker effect on self-efficacy than students’ own performance-based information (Schunk, 1991).

The third source, *social persuasion*, involves evaluative feedback from others and is based on the assumption that encouragement from others can enhance students’ beliefs in their capability to perform a given task at a certain level (Bandura, 1997). Several studies have shown a significant correlation between self-efficacy and social persuasion (e.g., Stevens et al., 2006; Usher and Pajares, 2009; Joët et al., 2011), but this source’s contribution to enhanced self-efficacy has been found to be temporary if a subsequent effort leads to poor results (Schunk, 1991). In light of social persuasion’s limited ability to create enduring improvements in self-efficacy, Bandura (1997) viewed it as a comparatively weak source. The final source, *physiological and affective states*, refers to the influence of anxiety, mood, stress and fatigue on self-efficacy beliefs (Bandura, 1997). For example, students with high anxiety levels may undermine their beliefs about their own abilities. Previous studies vary in their reports on the relationship between physiological and affective states and self-efficacy; for example, Stevens et al. (2006) and Usher and Pajares (2009) found significant correlations, while Joët et al. (2011) did not. Bandura (1997) viewed this particular source of self-efficacy information as the least influential, as it does not reliably diagnose capability.

According to Bandura (1997), self-efficacy is important because it influences motivational, decisional, cognitive and emotional processes. He asserted that a person with high self-efficacy would think more strategically and optimistically than a person with low self-efficacy. In addition, he found that self-efficacy influenced people’s choices, realisation of accomplishments, levels of stress and depression, effort, persistence, goals and achievement (Bandura, 2006). This has also been found in the body of literature reporting on self-efficacy in the context of learning mathematics, in which self-efficacy may influence task choice, effort, persistence, self-evaluation, resilience and achievement (Zimmerman and Martinez-Pons, 1990; Pajares and Miller, 1995; Pajares, 1996; Ramdass and Zimmerman, 2008; Schunk and Mullen, 2012; Zakariya, 2021), and is an even better predictor of achievement when students are accurate in judging their self-efficacy (Chen and Zimmerman, 2007).

When measuring self-efficacy, it is important to measure self-efficacy close in time to the given task (Bandura, 1997). Moreover, Bandura (2006) recommended not using a ‘one-measure-fits-all’ approach since it is often too general, but rather, to measure perceived self-efficacy as tailored to the object of interest. This is supported by several researchers who claim that, to increase prediction, measuring self-efficacy should be task-specific and measured before the task is performed (Pajares and Miller, 1995; Zakariya et al., 2019). While taking all these considerations into account, additionally, since mathematics self-efficacy is concerned with perceived capability, in the current study, we use the phrase “can do” instead of “will do”, as recommended by Bandura (2006). Bandura (1997) pointed out that “will” is about intention and is not a measure of a person’s judgement of their capabilities.

### 2.2. Differentiation in mathematics textbooks

As discussed in the introduction, mathematics textbooks hold a strong position as the main resource for planning and executing mathematics teaching (Robitaille and Travers, 1992; Howson, 1995; Stein et al., 2007; Jablonka and Johansson, 2010; Dolonen et al., 2016) and are known to be extensively used in mathematics education across the world (Glasnović Gracin, 2014). For example, in Glasnovic Gracin’s (2011) study, textbooks were found to have an important place in mathematics teaching and learning in lower secondary education; teachers used them extensively to prepare lessons, both for using the methodology presented and as the main source for students’ practice. However, another example from a study investigating education in Estonia, Finland and Norway indicated that “almost 45% of the teachers use the textbook simply as an exercise book” (Lepik et al., 2015, p. 129). These findings, in combination with the need for differentiation initiatives in mathematics teaching (Tomlinson, 2001), highlight the need to investigate differentiation in textbooks to determine whether they are doing the job.

Differentiation in mathematics varies between countries (Pepin and Haggerty, 2003; Howson, 2013). A comparative study of mathematics textbook use conducted by Pepin and Haggerty (2003) revealed how France, England and Germany approached differentiation differently. In France, teachers used the same textbook for all students of the same age. While the content of the lessons was the same, the tasks were differentiated, and the teachers were responsible for selecting tasks from the textbook for the different students according to their abilities. In England, students were divided into three groups according to ability; each group had their own books, with tasks adjusted to their level. In Germany, students were grouped into different school types based on their prior achievements in school. Approaches also varied between school types, as textbooks were used as a framework and support for learning in low-achieving students but were used to a lesser extent amongst high-achieving students. Accordingly, Pepin and Haggerty (2003) found that concerns related to differentiation differed amongst the three countries.

Similarly, Lepik et al. (2015, p. 142) found that textbooks were used quite differently in Estonia, Finland and Norway based on how teachers saw their endeavour to differentiate; in Norway, 64% of the teachers agreed that the tasks in the textbook were adapted to both weak and strong students, while only half of the Estonian teachers and 46% of the Finnish teachers agreed with this statement. Brändström (2005) also reported on the use of mathematics textbooks in Sweden and found that the textbooks themselves seemed to guide the differentiation. Students often started on the same page, which described the theory and presented a set of tasks, and then undertook a diagnostic test before being divided into different levels based on the results of the test. In summary, even if textbooks’ structures and teachers’ use of textbooks differ between countries, textbooks consistently play a significant role in differentiation initiatives. The body of literature seems to support Czeglédy and Szász (2005), who asserted that the appropriate use of textbooks supports differentiation.

In line with Glasnović Gracin (2014), who drew attention to the need for research on the content and structure of textbooks, we were unable to find research reporting on the composition of textbooks and the distribution of different content components (such as the proportion of level-marked tasks). Therefore, knowing that selecting tasks is an essential part of teachers’ interactions with mathematics textbooks (Matic and Glasnovic Gracin, 2016), the first author of this paper took a closer look at the three most commonly used lower secondary mathematics textbooks in Norway (Tesfamicael and Lundeby, 2019) and found that between 60% and 98% of the tasks in these textbooks were level-marked tasks. While this study was conducted more out of curiosity than for the purpose of research, the high proportion of level-marked tasks suggests that they are worthy of further investigation.

In this paper, we aim to investigate whether the use of level-marked tasks as a differentiation initiative affects students’ beliefs about their ability to accomplish a given task. Against this backdrop, this paper advances the following research question: To what extent does the level marking of mathematics tasks affect students’ self-efficacy?

## 3. Materials and methods

**Figure 1:**
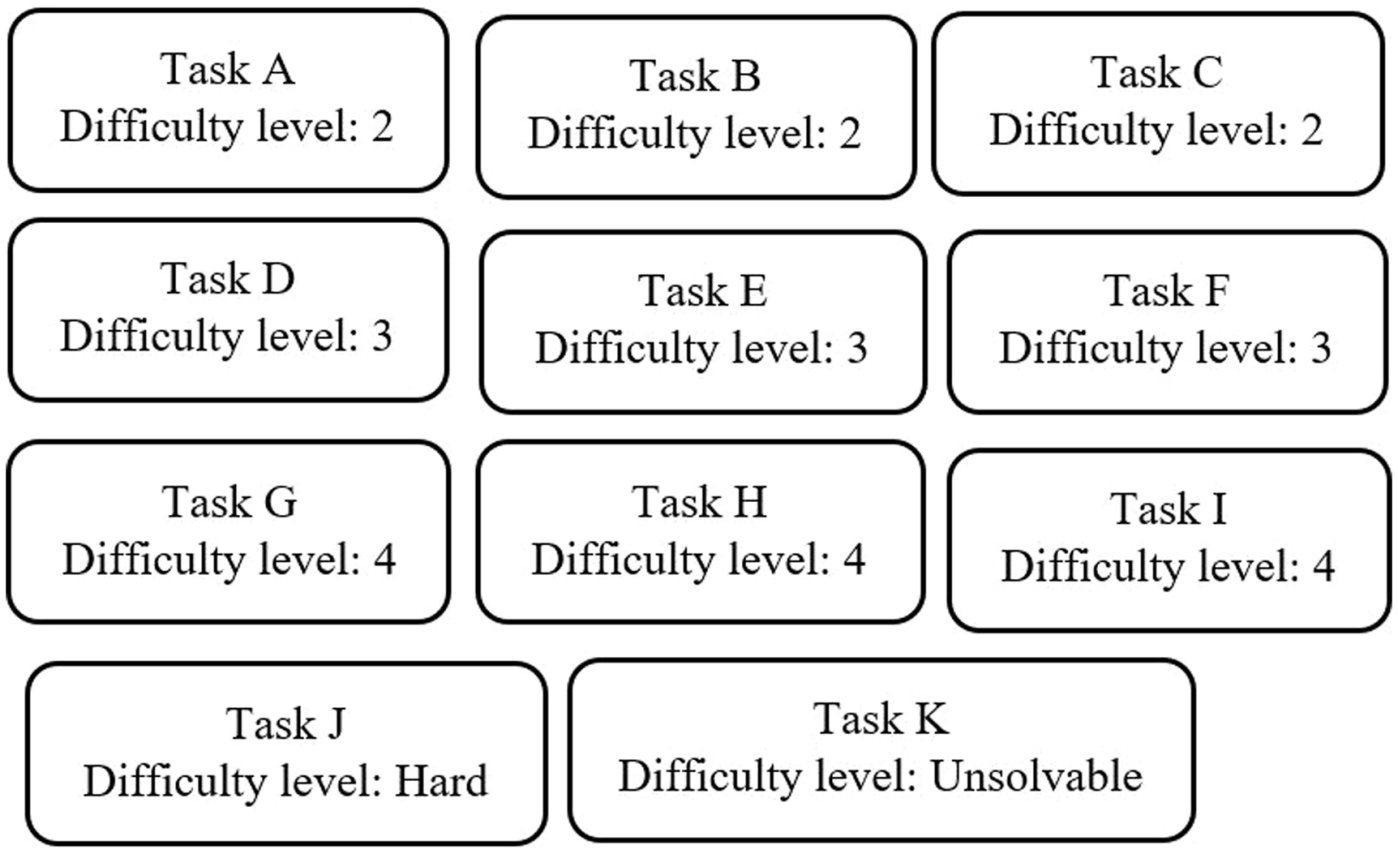
Difficulty levels for the 11 tasks in the larger project.

To investigate the effect of level-marked tasks on students’ self-efficacy, an online survey with a complex design was developed by the first author for a larger research project. The purpose of the larger project was to investigate the effect of level-marked tasks on students’ self-efficacy and to explore whether and how level marking affects motivational, decisional, cognitive and/or emotional processes. Hence, 11 tasks from the topic “arithmetic and algebra” formed the basis for an online survey. Of these, nine were retrieved from a national test in mathematics (Norwegian Directorate for Education and Training, n.d.), one was chosen, with some adjustment, from a mathematics website (Omtvei, n.d.), and one unsolvable task was created by the first author of this paper. The difficulty level of task A-I follows from the national test, and the difficulty level of Task J was marked as “hard” since only 17% of the students in a pilot study solved it correctly (Herset and El Ghami, 2022). Figure 1 illustrates the difficulty level of each of the 11 included tasks.

### 3.1. Selected tasks and design

To answer the research question in this paper, we analysed the responses given to Tasks A–C. They were chosen because they are similar in terms of difficulty level, topic and word length. This similarity is important when comparing students’ self-efficacy between tasks. To avoid a floor or ceiling effect (Everitt, 2002), it was important to choose tasks at an appropriate level—that is, tasks that were not too difficult or too easy. According to Björnsson (2016), 70% of Norwegian students are within the range of mastery levels 3–5, and 10% of students are at mastery level 1 in the national test in mathematics. For this reason, we chose tasks at mastery level 2 (Tasks A–C) for this study. The tasks are shown in Table 1. The students were asked to read the task and respond to the question, “How certain are you that you can solve this problem correctly?,” using a 100-point scale ranging from “Not certain at all” (0) to “Absolutely certain” (100), as recommended in the literature (Pajares et al., 2001; Bandura, 2006; Zakariya, 2019).

**Table 1:** The three selected tasks (authors’ translation).

Task A	In *Barcelona*, you find the not-yet-completed church known as the Sagrada Família. They started building it in 1882, and it is supposed to be finished in 2026. How many years do they expect it will take to build the Sagrada Família?
Task B	*Rita* is on holiday in *Greece*. She wants to rent a scooter. It costs NOK 25 per 5 min. How much does it cost to rent the scooter for 1 h?
Task C	*Silja* wants to take a swimming test. To do that, she has to swim 200 m without taking a break. The length of the pool is 12.5 m. How many lengths does *Silja* have to swim?

**Figure 2:**
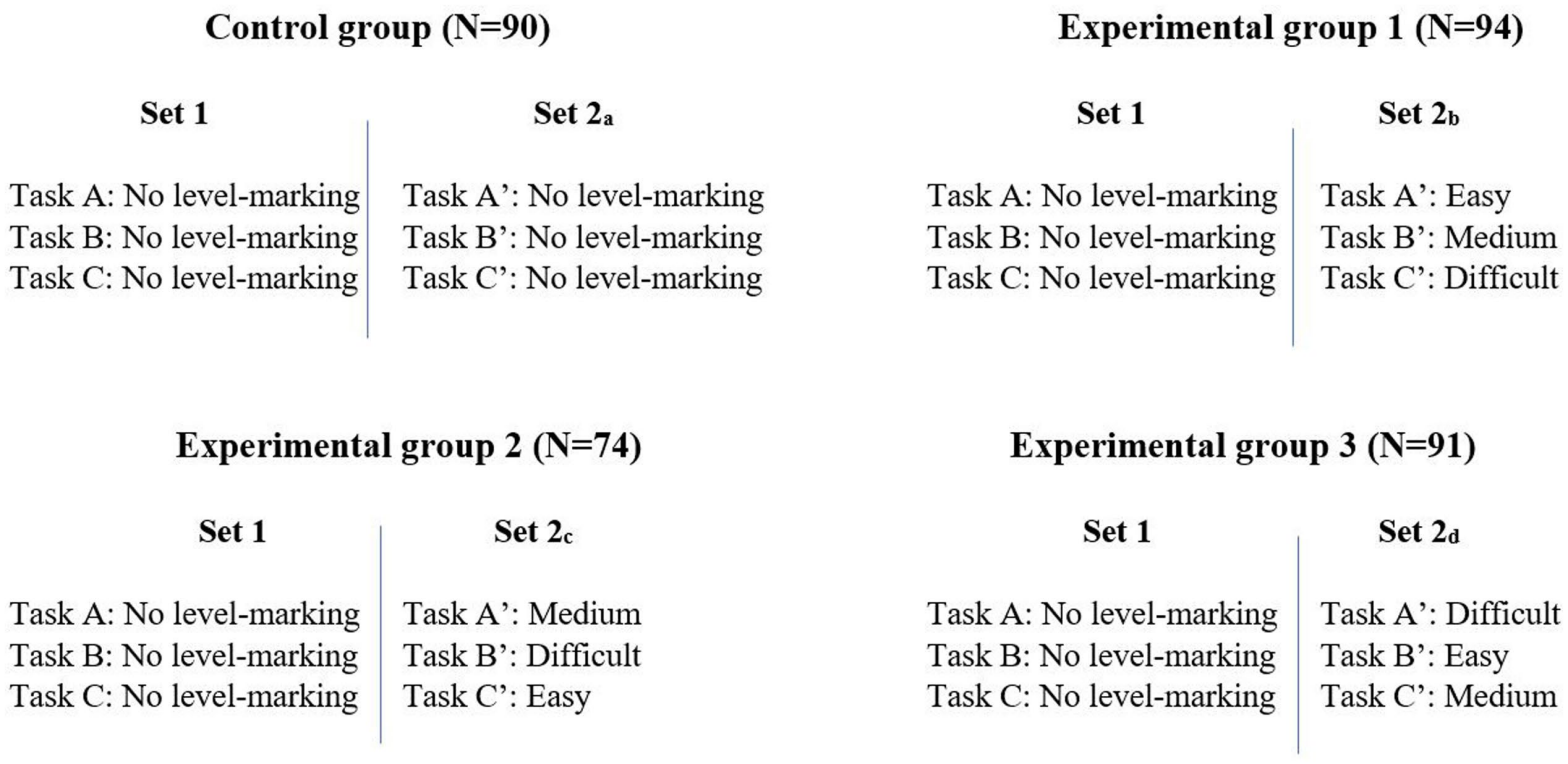
Outline of the design.

Because we utilised only selected parts of the collected data here, we describe only the aspects of the online survey that enabled us to gather these data. When students signed in to the survey, they were randomly assigned to one of four groups: the control group (CG) or to one of three experimental groups (EG_i_, i = 1, 2, 3). Once assigned to a group, the students received two sets of tasks (see Figure 2). Set 1 was identical for all four groups, while Set 2 was different in terms of the labelling of the tasks (and are labelled 2_a_, 2_b_, 2_c_, and 2_d_ accordingly).

In Set 1, none of the tasks were level marked. This was true for all four groups. In Set 2_a_, CG participants received Tasks A–C again and none of the tasks were level marked. In Sets 2_b_, 2_c_, and 2_d_, the students were presented with Tasks A–C again, but this time they were marked as “easy,” “medium” and “difficult,” and the marking changed between groups (see Figure 2). In all four editions of Set 2, to avoid the tasks being identical to Set 1, the words in italics in Table 1 were replaced to give the tasks a new “outlook” (e.g., in Task B, *Rita* was replaced with *Alex*, *Greece* was replaced with *France*, and *she* was replaced with *he*). As shown in Figure 2, we marked the tasks in Set 2 with an apostrophe (A′, B′ and C′) to illustrate that they got a new “outlook” without changing the content.

**Figure 3:**
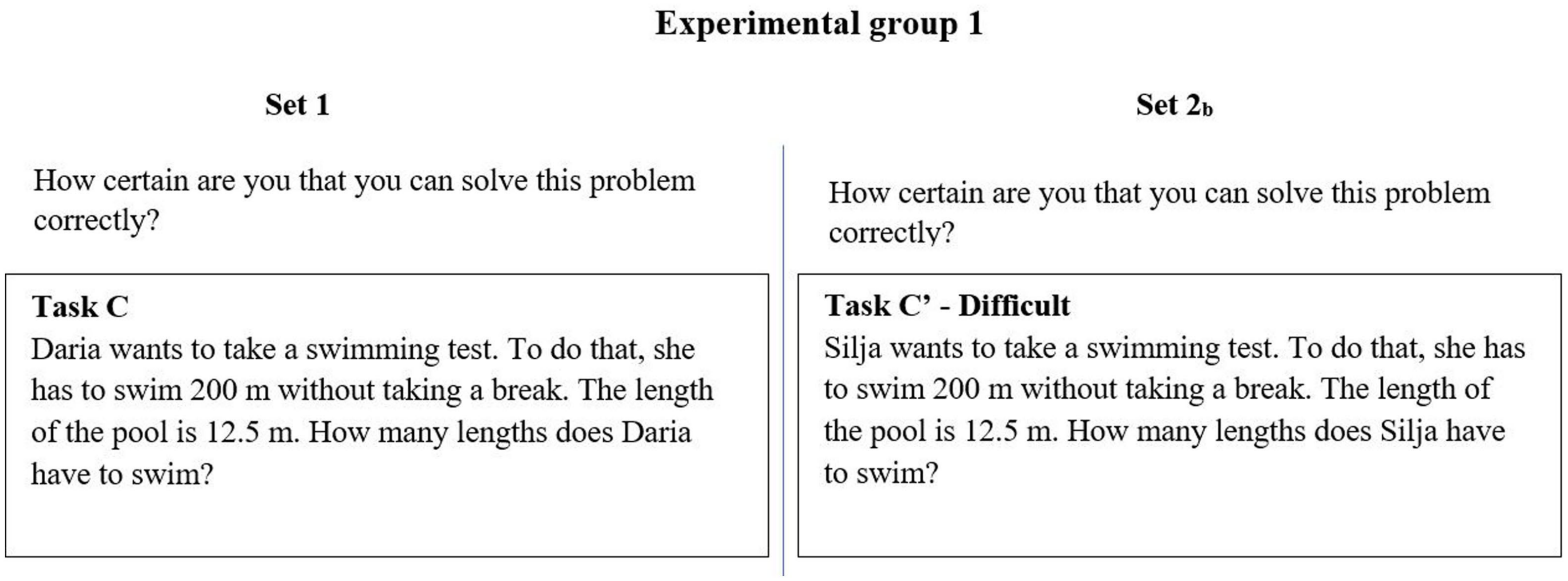
An example of how Task C appears for EG_1_ (translated by the first author).

To clarify the design, Figure 3 shows an example of how Task C appeared for EG_1_ in Set 1 and Set 2_b_. As shown, everything appears similar apart from the names (“Daria” and “Silja”) and in addition, in Set 2_b_, Task C′ is marked as “difficult”.

In this study, following Cohen et al. (2018), we viewed reliability as equivalence, consistency and stability. The design of our study enabled a comparison between how students responded to similar tasks, and even the same task, with and without level markings. The CG was included for reliability purposes only, and Wilcoxon tests revealed no significant change in self-efficacy scores between Set 1 and Set 2_a_ (both sets without level-marked tasks) for any of the tasks; thus, reliability as equivalence was considered to have been achieved. Reliability as consistency was tested in the CG, where a Friedman repeated test showed no significant difference (which was exactly what we wanted) when comparing students’ difference in self-efficacy (Set 2 – Set 1) between each of the three tasks A–C. We did not use the instrument repeatedly over time, so stability was not evaluated, which could be considered a limitation of our cross-sectional study.

### 3.2. Participants

Since the population is large and widely dispersed, we used cluster sampling (Cohen et al., 2018). After the first author had randomly chosen schools across Norway to participate in this study, students in grades 8 and 9 (i.e., aged 13–15 years) were recruited by first contacting the chosen schools’ principals. If they were willing to participate, they encouraged the school’s mathematics teachers to facilitate their students’ participation. Because of COVID-19, some of the randomly chosen schools were not able to participate and were replaced by other schools. The students responded to the survey during class, and the teachers made sure that the data were collected following a set of predetermined instructions (e.g., students shall not collaborate) and that ethical guidelines were followed (e.g., no student shall feel obligated to participate).

An analysis of missing patterns suggested that some of the data were incomplete or monotone, indicating that participants had skipped items; hence, 84 responses were removed. In addition, three response strings were detected as outliers, of which two were deleted because of extreme values and the third was removed because the participant spent an unrealistic amount of time on the survey. The final sample used in this analysis included *n* = 349 students, of which 172 (49.3%) were female and 177 (50.7%) were male, coming from 23 schools from all regions in Norway (47% from Northern Norway, 10% from Mid Norway, 9% from Western Norway, 4% from Southern Norway and 30% from Eastern Norway). The students were distributed as follows: *n* = 90 in CG, *n* = 94 in EG_1_, *n* = 74 in EG_2_, *n* = 91 in EG_3_ (see Figure 2).

### 3.3. Statistical methods

In response to the call by McNeill and Polly (2023) for more research examining the interaction between students’ self-efficacy and teachers’ differentiation initiatives, and in line with our research question, our data collection design enabled us to investigate both how and to what extent differentiation in the form of level-marked tasks affects students’ self-efficacy. The survey design allowed us to investigate how the different level markings of tasks affected students’ responses. Hence, we formulated the following two hypotheses:

*H1*: There is a gap in students’ self-efficacy when approaching the same tasks with and without level marking.

*H2*: The gap between students’ self-efficacy when encountering the same tasks with and without level marking expands with increasing difficulty markings.

The hypotheses are formulated in such a way that H2 makes sense only if our data support H1. To test H1 and H2, we merged all student responses to easy-marked tasks and did the same for medium-marked and difficult-marked tasks. H1 was tested by comparing the medians of students’ self-efficacy scores when receiving the same task with and without level marking. We used two-tailed test as suggested by Cohen et al. (2018) because the non-directional hypothesis indicates only difference, and not whether self-efficacy would be positively or negatively affected by level-marked tasks. Because the data were nonparametric, we used a series of Wilcoxon tests. To test H2, we used the Friedman test to check whether the difference in students’ self-efficacy when receiving tasks with and without level markings was significantly different between easy-, medium- and difficult-marked tasks.

The overall project was given full ethics approval by the Norwegian Social Science Data Service, ensuring the interests of the participants. We are aware of the limitations of this study, which are mainly connected to the small sample size and skewed distribution of the participating schools across Norway. We are mindful of the limits on the generalisability of our results.

## 4. Results

Our research question and associated hypotheses were formulated on the basis of the reviewed literature. Taken together, if both hypotheses held, we would have an argument for the effect of level-marked mathematics tasks on students’ self-efficacy. Descriptive statistics related to tasks A, B and C are presented in Table 2.

**Table 2:** Students’ self-efficacy in Set 1 and Set 2.

	Set 1				Set 2				
		Median	Mean	SD	Median	Mean	SD
Task A	EG_1_	100.00	87.89	19.94	EG_1(Easy)_	99.50	85.11	21.48
	EG_2_	98.50	87.09	20.74	EG_2(Medium)_	96.50	86.24	21.33
	EG_3_	97.00	85.76	21.48	EG_3(Difficult)_	95.00	82.15	23.08
Task B	EG_3_	100.00	86.90	21.22	EG_3(Easy)_	100.00	86.08	20.28
	EG_1_	97.50	86.26	19.56	EG_1(Medium)_	94.50	83.53	20.28
	EG_2_	99.00	88.19	20.40	EG_2(Difficult)_	95.00	84.32	23.00
Task C	EG_2_	90.00	79.38	25.73	EG_2(Easy)_	92.00	80.50	26.09
	EG_3_	88.00	80.65	20.91	EG_3(Medium)_	90.00	80.54	22.31
	EG_1_	90.00	78.22	25.88	EG_1(Difficult)_	80.00	73.25	27.16

EG_1-3_: Tasks without level marking (Set 1). EG_1-3(easy/medium/difficult)_: Tasks with level markings (Set 2).

When comparing the two “mean” columns (columns 4 and 8) and the two “median” columns (columns 3 and 7) in Table 2, we see how reported self-efficacy declines as tasks go from no level marking to being marked as difficult.

Because of the way in which this study was designed, all students in the EGs received the three similar tasks twice. This means that all students, regardless of which EG they were in, received three tasks in Set 2 with different level markings: easy, medium and difficult (see Figure 2 in the methods section). Hence, we had 259 student responses (i.e., one response from each of the [94 + 74 + 91] students in all three EGs) to easy-marked tasks, medium-marked tasks and difficult-marked tasks. This enabled us, in hypothesis testing, to examine the differences in self-efficacy of the responses between no level marking and easy-level marking, between no level marking and medium-level marking, and between no level marking and difficult-level marking. We found that the effect of difficult-level marking was the largest, as illustrated in Table 3.

**Table 3:** Mean difference in students’ self-efficacy between Set 2 (easy-, medium- and difficult-level marking) and Set 1 (without level marking).

Set 2 – Set 1	Mean difference in students’ self-efficacy
Easy-level marking – Without level marking	−0.98
Medium-level marking – Without level marking	−1.27
Difficult-level marking – Without level marking	−4.18

As the same students’ in the EGs answered Sets 1 and 2, the sample is dependent, and the Wilcoxon test was used because the data were not normally distributed (Cohen et al., 2018). As shown in Table 4, a Wilcoxon test revealed that students’ self-efficacy was significantly lower when tasks were marked as difficult, *z* = −4.033, *p* < 0.001. There was no significant difference between no level marking and medium-level marking (*z* = −0.930, *p* = 0.353) or between no level marking and easy-level marking (*z* = −0.233, *p* = 0.824).

**Table 4:** Wilcoxon test of the difference in students’ self-efficacy (Set 2–Set 1).

		*N*	Mean rank	Sum of ranks	*Z*	Two-tailed value of *p*
Set 2 (easy-level marking) – Set 1 (without level marking)	Negative ranks	61^a^	74.66	4554.50	−0.233^d^	0.824
	Positive ranks	72^b^	60.51	4356.50		
	Ties	126^c^				
	Total	256				
Set 2 (medium-level marking) – Set 1 (without level marking)	Negative ranks	79d^a^	79.99	6319.00	−0.930^d^	0.353
	Positive ranks	73^b^	72.73	5309.00		
	Ties	107^c^				
	Total	259				
Set 2 (difficult-level marking) – Set 1 (without level marking)	Negative ranks	101^a^	88.19	8907.50	−4.033^d^	<0.001**
	Positive ranks	60^b^	68.89	4133.50		
	Ties	98^c^				
	Total	259				

**The difference is significant at the 0.01 level.

a Set2 < Set1.

b Set2 > Set1.

c Set2 = Set1.

d Based on positive ranks.

To test H2—that is, to determine whether the differences highlighted in Table 3 were statistically significant—Friedman tests were carried out (see Table 5). This revealed a significant effect of the level marking on students’ self-efficacy, *χ*^2^ (2, *n* = 259) = 11.413, *p* = 0.003, <0.01. The medians indicated that students’ differences in self-efficacy were highest when the tasks were marked as difficult, followed by medium- and easy-level marking.

**Table 5:** Friedman test.

	*N*	Mean rank	*χ* ^2^	df	Value of *p*
Easy	259	1.90	11.413	2	0.003**
Medium	259	1.96			
Difficult	259	2.14			

**The difference is significant at the 0.01 level.

Further analyses with Friedman tests were conducted to follow up pairwise comparisons. These pairs were set up in the following manner: Pair 1 compared *x* and *y*, where *x* is the difference in median between “self-efficacy with no level marking” and “self-efficacy with easy-level marking” and *y* is the difference in median between “self-efficacy with no level marking” and “self-efficacy with medium-level marking.” In the same manner, Pair 2 dealt with students’ responses to medium- and difficult-level marked tasks and Pair 3 with easy- and difficult-marked tasks (see Table 6).

**Table 6:** Pairwise comparisons.

	Level marking	*N*	Test statistic	Std. error	Two-tailed value of *p*
Pair 1	Easy	259	−0.066	0.088	0.455
	Medium	259			
Pair 2	Medium	259	−0.176	0.088	0.046*
	Difficult	259			
Pair 3	Easy	259	−0.241	0.088	0.006**
	Difficult	259			

*The difference is significant at the 0.05 level. **The difference is significant at the 0.01 level.

Overall, the results in Table 6 show that the effect on students’ self-efficacy was significant when testing Pair 2 (going from no level marking to difficult-marked tasks, compared to going from no level marking to medium-marked tasks; *p* = 0.046), and Pair 3 (going from no level marking to difficult-marked tasks, compared to going from no level marking to easy-marked tasks; *p* = 0.006). The trend also applied in testing Pair 1 (going from no level marking to medium-marked tasks, compared to going from no level marking to easy-marked tasks), but this difference was not significant (*p* = 0.455). However, the effect on students’ self-efficacy was significantly larger when going from no level marking to difficult-marked tasks, compared to going from no level marking to easy- and medium-marked tasks. Taken together, this shows that the gap between students’ self-efficacy when encountering the same tasks with and without level marking expands going from easy- to difficult-marked tasks and from medium to difficult-marked tasks.

## 5. Discussion and concluding remarks

When encountering a mathematics task, most people are affected by additional information, such as information about the task’s level of difficulty. The most striking result from our analysis was the extent to which tasks marked as difficult had a negative effect on students’ self-efficacy. We found that students reported a significantly lower level of self-efficacy when encountering tasks marked as difficult compared to when they encountered the same task without level marking. Further, the difference in students’ self-efficacy when solving tasks with and without level marking became larger when the markings denoted increasing difficulty levels. Here, we discuss what this finding means for students in terms of their mathematics learning and what it means for mathematics teachers’ differentiation initiatives and for future mathematics textbooks.

Whether a student perceives a given task as being easy or difficult is a matter of personal opinion. This affects the student’s level of self-efficacy, which in turn influences the strength of their self-efficacy (Bandura, 1997). The negative effect of difficult-level markings on students’ self-efficacy highlights that even when all students receive the same task, the expectation of mastery becomes lower when a task is marked as difficult. This is consistent with Street et al.’s (2017) finding that students’ perceptions of difficulty could be different from the actual difficulty level. When tasks were marked as easy, this did not affect students’ self-efficacy, which suggests that the students did not perceive the tasks to be any easier than when no level markings were given. Keeping in mind that the first author designed the study using easy tasks—at mastery level 2 of 5 (Björnsson, 2016)—an effect for easy marking might have arisen if the focus had been on tasks with a higher difficulty level. More research is required to determine how level markings affect different levels of actual difficulty.

Although the sources of self-efficacy were not directly measured in this study, the results of our study apply to this body of research. As reported in previous research, mastery experience is the most powerful source of self-efficacy (Bandura, 1997; Stevens et al., 2006; Usher and Pajares, 2009; Joët et al., 2011; Butz and Usher, 2015), and this is a good reason for believing that some of the students’ previous mastery experiences with difficult-marked tasks had affected their self-efficacy negatively. This is in line with Skaalvik and Skaalvik (2018, p. 197), who claimed that mastery experiences are necessary for students to develop and preserve expectations of mastery. A possible interpretation of this finding is that level marking affects students’ perceptions of the level of difficulty, and if their mastery experience has previously been low when solving tasks marked as difficult, their level of self-efficacy may decrease. This resonates with Bandura (1997), Chen and Zimmerman (2007), and Street et al. (2022b), who suggested that students’ opinions about whether tasks are easy or difficult affect their self-efficacy.

Our results can also be attributed to students’ physiological and affective states, in that their self-efficacy beliefs are informed by anxiety, mood, stress and fatigue (Bandura, 1997). When told that a task is difficult, some draw on this comparatively weak source of self-efficacy, with detrimental results. This could explain some of the negative effects we found. No positive effects of level marking were found, which seems to indicate that level marking does not improve students’ physical or emotional well-being. However, previous studies are inconsistent in their conclusions on this point; for example, Stevens et al. (2006) and Usher and Pajares (2009) found significant correlations between self-efficacy and physiological and affective states, while Joët et al. (2011) did not. In terms of the last two sources of self-efficacy—social persuasion and vicarious experiences—we could only speculate about how they may have affected our results. Qualitative research is required to investigate this in greater detail.

Surprisingly, no positive effect of level marking on students’ self-efficacy was found. However, the present study did not investigate how level marking affects students with different self-efficacy strengths. It is likely that the effect of level marking is different for groups of students with high and low self-efficacy. This was supported by Schunk (1991), who claimed that a person with a high sense of self-efficacy would be more motivated, persist longer and be willing to expend a higher degree of effort. Further research is required to determine exactly how the effect of level marking on students’ self-efficacy varies by strength of self-efficacy, as well as how the effect of level marking varies between groups of students (e.g., according to gender, grade, motivational factors and mastery experiences).

We are aware that our research may have some limitations related to the voluntary nature of participation in the survey, sample size and data collection taking place *via* the schools’ principals. We attempted to select schools randomly, but because several schools withdrew due to COVID-19, we had to choose several schools in one district to obtain sufficient data. Moreover, in Norway, there are ~113,700 students in grades 8 and 9, and our data collection consists of *n* = 436 students. On the one hand, according to the sample size table (Cohen et al., 2018, p. 207), a sample of 383 students is recommended, which is lower than the number of participants in our study (*n* = 436). On the other hand, the participants were divided into different groups and there were missing data, so the sample size might be a limitation. Moreover, due to COVID, surveys were distributed to students by their teacher, which limited our opportunities to ensure sufficiently good and purposeful data collection. These limitations highlight the difficulty of collecting data, especially during COVID.

In reviewing the literature, we found that some countries, such as England and Germany, utilise mathematics textbooks that are adapted to different levels of ability (Pepin and Haggerty, 2003), indicating that level-marked tasks may not appear consistently in English and German mathematics classrooms. However, the use of level-marked tasks is extensive in Norwegian mathematics textbooks (Grave and Pepin, 2015) and classrooms (Eriksen et al., 2022). Although Skaalvik and Fossen (1995) claimed that textbooks require differentiation, our finding that the level marking of tasks negatively affects students’ self-efficacy suggests that there is a need to investigate this in more detail. Brändström (2005) raised questions regarding the level marking of tasks in Swedish mathematics textbooks nearly two decades ago, and to our knowledge, nothing has changed since then.

Glasnović Gracin (2014) highlighted the need for research on the content and structure of textbooks. We add to this call by pointing the research path in the direction of level-marked tasks, specifically in terms of the number of such tasks in textbooks, their stated purpose as specified by textbook authors and how they are intended to contribute to better learning. The findings of the current study show that the level marking of tasks appears to have a detrimental effect on students’ beliefs in their own ability to accomplish the tasks. Finding that difficult-level-marked mathematics tasks may result in reduced self-efficacy in students may indicate that marking tasks as difficult has consequences for students’ learning. The level marking of tasks may result in students’ avoidance of difficult tasks and lead to low and inaccurate self-efficacy judgements, which can in turn affect their achievement. This negative effect on students’ self-efficacy is the opposite of what level marking is intended to achieve.

Our results contribute to a new understanding of level-marked tasks in mathematics textbooks as a differentiation initiative. The results indicate that level marking does not improve self-efficacy, which contradicts the purpose of differentiation (Mathiassen, 2009; NOU, 2016, p. 62). The finding that difficult-level marking of tasks reduces students’ self-efficacy has implications for mathematics teachers in terms of their choice of differentiation initiatives. This study adds new insights to the body of research reporting on how self-efficacy affects task choice, effort, persistence, self-evaluation, resilience and achievement (Zimmerman and Martinez-Pons, 1990; Pajares and Miller, 1995; Pajares, 1996; Ramdass and Zimmerman, 2008; Schunk and Mullen, 2012; Zakariya, 2021), and may have implications for how teachers use level-marked tasks in the classroom. If teachers allow students to choose between level-marked tasks, a negative consequence might be that some students avoid tasks marked as difficult. However, considering that the present study investigated only three tasks, more research is required to determine how level-marked tasks affect students’ cognitive, affective, selective and motivational processes. In addition, we recommend that future research include more than one mathematics task per level to measure the internal consistency of students’ self-efficacy. We are currently in the process of investigating the effect of level-marked tasks on students’ performance, persistence and choice of tasks, for a future examination of how level-marked tasks affect students’ learning of mathematics.

## Data availability statement

The raw data supporting the conclusions of this article will be made available by the authors, without undue reservation.

## Ethics statement

The studies involving human participants were reviewed and approved by NSD – Norwegian centre for research data. Written informed consent to participate in this study was provided by the participants’ legal guardian/next of kin.

## Author contributions

All authors listed have made a substantial, direct, and intellectual contribution to the work and approved it for publication.

## Conflict of interest

The authors declare that the research was conducted in the absence of any commercial or financial relationships that could be construed as a potential conflict of interest.

## Publisher’s note

All claims expressed in this article are solely those of the authors and do not necessarily represent those of their affiliated organizations, or those of the publisher, the editors and the reviewers. Any product that may be evaluated in this article, or claim that may be made by its manufacturer, is not guaranteed or endorsed by the publisher.
